# Human telomerase reverse transcriptase positively regulates mitophagy by inhibiting the processing and cytoplasmic release of mitochondrial PINK1

**DOI:** 10.1038/s41419-020-2641-7

**Published:** 2020-06-08

**Authors:** Woo Hyun Shin, Kwang Chul Chung

**Affiliations:** Department of Systems Biology, College of Life Science and Biotechnology, Yonsei University, 03722 Seoul, Korea

**Keywords:** Mitophagy, Proteolysis

## Abstract

Mutations in the phosphatase and tensin homologue-induced putative kinase 1 (PINK1) gene have been linked to an early-onset autosomal recessive form of familial Parkinson′s disease (PD). PINK1, a mitochondrial serine/threonine-protein kinase, plays an important role in clearing defective mitochondria by mitophagy – the selective removal of mitochondria through autophagy. Evidence suggests that alteration of the PINK1 pathway contributes to the pathogenesis of PD, but the mechanisms by which the PINK1 pathway regulates mitochondrial quality control through mitophagy remain unclear. Human telomerase reverse transcriptase (hTERT) is a catalytic subunit of telomerase that functions in telomere maintenance as well as several non-telomeric activities. For example, hTERT has been associated with cellular immortalization, cell growth control, and mitochondrial regulation. We determined that hTERT negatively regulates the cleavage and cytosolic processing of PINK1 and enhances its mitochondrial localization by inhibiting mitochondrial processing peptidase β (MPPβ). Consequently, hTERT promotes mitophagy following carbonyl cyanide *m*-chlorophenylhydrazone (CCCP)-induced mitochondrial dysfunction and improves the function of damaged mitochondria by modulating PINK1. These findings suggest that hTERT positively regulates PINK1 function, leading to increased mitophagy following mitochondrial damage.

## Introduction

Parkinson′s disease (PD) is a neurodegenerative disease that predominately affects dopaminergic neurons in a specific area of the midbrain^1^. The cause of PD remains unclear, but several gene mutations related to familial PD have been identified^2^. Among the mutations, various mutations in *the phosphatase and tensin homologue-induced putative kinase 1* (PINK1) and *parkin* genes have been associated with an early-onset form of PD. Increasing evidence suggests that mitochondrial dysfunction plays a central role in the pathogenesis of PD, and that the subsequent activation of PINK1 and parkin plays a critical role in mitochondrial quality control^3^. PINK1 synthesized de novo in the cytosol is then rapidly and constitutively imported into the mitochondria by translocase of the outer membrane (TOM) and translocase of the inner membrane (TIM) complexes^4^. Once in the mitochondria, PINK1 is cleaved by mitochondrial processing peptidase (MPP) and presenilin-associated rhomboid-like protease (PARL)^5,6^, and the processed form of PINK1 is transported back to the cytosol and degraded rapidly by the proteasome^7,8^.

Treatment with carbonyl cyanide *m*-chlorophenyl hydrazone (CCCP), a mitochondrial uncoupler that induces mitochondrial depolarization, leads to the accumulation of PINK1 on the outer mitochondrial membrane and the phosphorylation of the Ser65 residue of ubiquitin (Ub) and parkin, an Ub E3 ligase^9,10^. Phosphorylated parkin is subsequently recruited to the mitochondria, where it attaches to the Ub chains of the substrate protein present on the mitochondrial surface. Autophagy adapters then bind to phospho-Ub at Ser65 and the autophagic machinery facilitates lysosomal degradation of the damaged mitochondria through mitophagy^11^. Importantly, the precise regulation of PINK1 processing is critical to mitophagy, and the accumulation of PINK1 in the mitochondrial outer membrane in response to various internal and exterior stimuli that cause the mitochondrial damage, including oxidative stress or the loss of mitochondrial membrane potential, is specifically required.

Ribonucleoprotein telomerase, which is essential for maintaining telomere length, is composed of multiple protein subunits, including human telomerase reverse transcriptase (hTERT), a catalytic protein subunit of telomerase, telomerase RNA (TR), an RNA component that serves as a template for elongation of the telomeric end, and telomerase-associated proteins^12^. In addition to its regulatory role in telomeres, hTERT functions in other non-telomeric activities such as cell immortalization, DNA repair, and cell growth control^13^. In particular, hTERT reportedly alleviates intracellular production of reactive oxygen species (ROS), improving mitochondrial function and inhibiting ROS-mediated apoptosis^14^. Notably, ROS is a primary cause of oxidative stress and loss of mitochondrial membrane potential in cells.

Based on prior findings that chaperone complexes, including Hsp90, bind to and regulate the activities of PINK1 and hTERT, respectively, and that ROS-mediated mitochondrial dysfunction are alleviated by these proteins^15–17^, we hypothesized that a functional relationship existed between hTERT and PINK1 within the mitochondria. To verify our hypothesis, we investigated the potential effects of hTERT on PINK1-mediated mitophagy. Our results demonstrated that hTERT increases mitophagy by decreasing the processing of PINK1, resulting in the accumulation of full-length PINK1 on the outer mitochondrial membrane. We also determined that hTERT binds to the β subunit of MPP (MPPβ) and inhibits the cleavage of PINK1 through MPP, which might be the mechanism underlying the hTERT-induced suppression of PINK1 processing within the mitochondria and its subsequent release into the cytosol. Although treatment with anisomycin, a eukaryotic protein synthesis inhibitor, reportedly increases intracellular hTERT levels in cells^18^, its effect on PINK1 accumulation occurs via hTERT involvement. Moreover, the binding of PINK1 to the TOM complex, followed by parkin was likewise increased by hTERT. Collectively, the results that hTERT controls mitophagy by modulating PINK1.

## Materials and methods

### Materials

Dulbecco’s Modified Eagle Medium (DMEM), fetal bovine serum (FBS), and Lipofectamine and PLUS reagents were purchased from Invitrogen (Carlsbad, CA, USA). Protein A-Sepharose beads were obtained from GE Healthcare Life Sciences (Piscataway, NJ, USA). Anti-hemagglutinin (HA), anti-c-Myc, anti-tubulin, anti-actin, anti-GFP, anti-Tom20, and anti-Hsp90 antibodies were purchased from Santa Cruz Biotechnology (Santa Cruz, CA, USA). Polyclonal anti-PINK1 antibody was purchased from Novus (Littleton, CO, USA). Polyclonal anti-hTERT antibody was purchased from Genetex (Irvine, CA, USA). Polyclonal anti-parkin and anti-BNIP3L antibodies were purchased from Abcam (Cambridge, UK). Polyclonal anti-VDAC and anti-LC3 antibodies were purchased from Cell Signaling (Danvers, MA, USA). Anti-FLAG antibody was purchased from Sigma-Aldrich (St. Luis, MO, USA). Peroxidase-conjugated goat anti-rabbit and anti-mouse secondary antibodies were purchased from Millipore (Billerica, MA, USA). Alexa Fluor® 405-conjugated anti-mouse, Alexa Fluor® 488-conjugated anti-mouse and Alexa Fluor® 594-conjugated anti-rabbit secondary antibodies were purchased from Invitrogen. Enhanced chemiluminescence (ECL) reagent was purchased from Abclon (Seoul, Korea) and Advansta (Menlo Park, CA, USA). MG132 was purchased from A. G. Scientific (San Diego, CA, USA). All other chemicals used in the study were analytical grade commercial products and were purchased from Sigma- Aldrich.

### DNA constructs and RNA interference

The mammalian construct encoding Myc-tagged human wild-type PINK1 (pBOS-3×-Myc-hPINK1-WT) was kindly provided by J. Chung (Seoul National University, Seoul, Korea), and the plasmid encoding hTERT-HA was generated as previously described^19^. The cDNA encoding FLAG-tagged MPPβ was PCR-amplified using the following primers: forward primer, 5′-AGACGCGTATGGCGGCTGCGGC-3′, and reverse primer, 5′-AGGCGGCCGCTTAATCACGAAGCCAACACATGT-3′. The PCR product was then subcloned into a pRK5-FLAG vector. All cDNA sequences were verified by sequencing (BIONICS, Seoul, Korea). The *hTERT*-specific and scrambled small interfering RNAs (siRNAs) were designed and synthesized by Bioneer (Seoul, Korea). The *hTERT*-specific siRNA duplex sense and antisense sequences were 5′-UGAUUUCUUGUUGGUGACAdTdT-3′ and 5′-UGUCACCAACAAGAAAUCAdTdT-3′, respectively. The *PINK1*-specific siRNA duplex sense and antisense sequence were 5′-GAAAUCCGACAACAUCCUUUUdTdT-3′ and 5′-AAAAGGAUGUUGUCGGAUUUCdTdT-3′, respectively.

### Cell culture and preparation of cell lysates

Mouse embryonic fibroblasts (MEFs) derived from PINK1-null (*PINK1*−/−) and control (*PINK1*+/+) or hTERT-null (*hTERT*−/−) and control (*hTERT*+/+) mice were provided by J. Shen (Harvard Medical School, Boston, MA, USA) and H. W. Lee (Yonsei University, Seoul, Korea), respectively. Human embryonic kidney 293 (HEK293) cells and human neuroblastoma SH-SY5Y cells were purchased from ATCC (Manassas, Virginia, USA). Cells were maintained in DMEM containing 10% FBS and 100 U/ml penicillin–streptomycin and grown at 37 °C in 5% CO_2_. All DNA transfections were performed using Lipofectamine and PLUS™ Reagent according to the manufacturer’s protocol. The cells were rinsed twice with ice-cold phosphate-buffered saline (PBS) and lysed in lysis buffer containing 50 mM Tris (pH 7.4), 1.0% Nonidet P-40, 150 mM NaCl, 10% glycerol, and the protease inhibitor cocktail including 1 mM Na_3_VO_4_, 1 µg/ml leupeptin, 1 µg/ml aprotinin, 10 mM NaF, and 0.2 mM phenylmethylsulfonyl fluoride.

### Co-immunoprecipitation and immunoblot analysis

Cell lysates containing ~500 μg protein were incubated overnight at 4 °C with 0.5 μg of the appropriate antibody. Samples were incubated with Protein A-Sepharose beads for 2 h at 4 °C with rotation. The beads were then pelleted and washed five times with lysis buffer. The immunocomplexes were dissociated by boiling in SDS–PAGE sample buffer, resolved by SDS–PAGE, and transferred to the nitrocellulose membranes. Membranes were blocked in Tris-buffered saline with Tween 20 (TBST) buffer containing 50 mM Tris (pH 7.4), 150 mM NaCl, 0.1% Tween 20, and 5% nonfat dry milk for 1 h at room temperature, and then incubated with the primary antibodies overnight at 4 °C in 3% nonfat dry milk. The membranes were then washed with TBST, incubated for 1 h with horseradish peroxidase (HRP)-coupled secondary IgG, washed again with TBST, and visualized using ECL reagents (Abclon, Seoul, Korea). Grayscale images of immunoblotting data were quantified by MultiGauge V. 3.1 program (Fujifilm Life Science, Tokyo, Japan). All target and loading control were validated in the linear range. The combined linear range was used to determine amount of sample loading. The band intensity was detected within combined linear range to perform quantitative western blot analysis.

### Immunocytochemistry analysis

The cells were seeded onto poly-l-lysine coated glass cover slips. After DNA transfection for 24 h, the cells were washed twice with PBS (pH 7.4) and immediately fixed in 3.7% formaldehyde for 10 min at room temperature. After fixation, the cells were permeabilized with 0.2% Triton X-100 for 10 min and blocked with 1% bovine serum albumin in PBS for 1 h at room temperature. The cells were then immunostained with the appropriate primary antibody, and Alexa Fluor™ 488-conjugated anti-mouse and Alexa Fluor™ 594-conjugated anti-rabbit secondary antibodies were used to detect the primary antibodies. Images were captured using a LSM 700 laser scanning confocal microscope (Carl Zeiss, Oberkochen, Germany), and data were processed using a Zeiss LSM Image Browser (Carl Zeiss). To determine the mitochondrial localization of target protein, cells were incubated with MitoTracker Red CMXRos (Invitrogen) for 1 h, washed with PBS, and visualized by confocal microscopy. To quantitatively measure the colocalization of PINK1, hTERT, and mitochondrial markers, analysis of Pearson’s correlation coefficient was performed by Image J software (National Institutes of Health, USA).

### Analysis of mitochondrial membrane potential

The mitochondrial membrane potential (ΔΨm) of the cells was analyzed using JC-1 dye (Life Technologies) according to the manufacturer’s instructions. Briefly, the cells were stained with 0.5 μg/ml JC-1 for 20 min at 37 °C in 5% CO_2_. The cells were then washed three times with PBS (pH 7.4) and analyzed using the LSM 700 confocal microscope (Carl Zeiss). The orange- and green-fluorescent signal indicated the cells with hyperpolarized and depolarized membrane potentials, respectively.

### Determination of intracellular ATP level

Intracellular ATP concentrations in the cells were measured using an ATP determination kit (Invitrogen) containing recombinant firefly luciferase and its substrate D-luciferin according to the manufacturer’s instruction.

### Statistical analysis

All statistical analyses were performed using an unpaired Student’s *t* test and the IBM SPSS statistical analysis software (version 23.0). All values are expressed as the mean ± standard error of the mean (SEM). Sample size was determined based on the previous studies with similar experiments (*n* number noted in the specific figure legends).

## Results

### PINK1 interacts with hTERT in mammalian cells

**Fig. 1:**
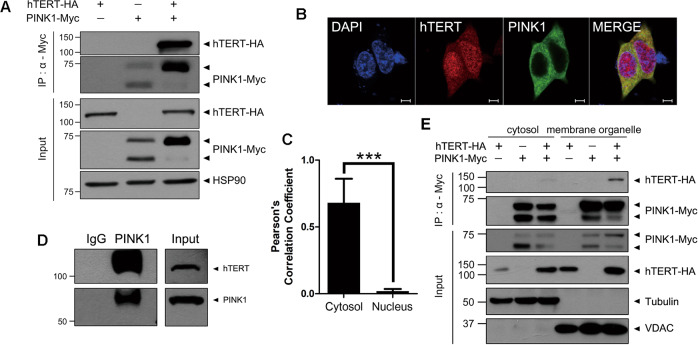
PINK1 binds to hTERT. **a** HEK293 cells were transfected with a plasmid encoding Myc-PINK1 and/or hTERT-HA for 48 h. Total cell lysates were immunoprecipitated with anti-Myc antibody and immunoblotted with the indicated antibodies. Hsp90 served as a loading control. **b** Representative confocal images of endogenous PINK1 (green) and hTERT (red) immunostaining are shown. Scale bar = 5 μm. **c** Pearson′s correlation coefficient of the colocalization between PINK1 and hTERT in Fig. 1b was analyzed by Image J software. Data are presented as the mean ± SEM of three independent experiments (***p ≤ 0.001). **d** SH-SY5Y cell lysates were immunoprecipitated with anti-PINK1 IgG and immunoblotted with the indicated antibodies. The cell lysates were immunoprecipitated with pre-immune IgG as a negative control. **e** HEK293 cells were transfected for 48 h with a plasmid encoding Myc-PINK1 and/or hTERT-HA, and the resulting cell lysates were separated into cytosolic and membrane organelle fractions. The samples were then immunoprecipitated with anti-Myc antibody and immunoblotted with the indicated antibodies. Tubulin and VDAC served as markers for the cytosolic and the mitochondrial fractions, respectively.

Based on prior evidence of the non-telomeric and mitochondria-related functions of hTERT, including the modulation of mitochondrial function and the reduction of intracellular ROS^14^, we examined the biochemical and functional interaction between hTERT and PINK1, and sought to determine if hTERT affected PINK1-mediated mitophagy. To first determine if hTERT binds to PINK1 in mammalian cells, we co-immunoprecipitated (co-IP) cell lysates transfected with a plasmid encoding Myc-tagged PINK1 alone or with a plasmid encoding HA-tagged hTERT. The results of immunoblot analyses revealed that ectopically expressed PINK1 binds to hTERT in HEK293 cells (Fig. 1a). In addition, immunocytochemical analyses of HEK293 cells revealed that endogenous hTERT and PINK1 colocalize, primarily outside the nuclei, with the value of Pearson′s correlation coefficient 0.68 (Fig. 1b, c). The interaction between endogenous PINK1 and hTERT was further confirmed in SH-SY5Y cells (Fig. 1d).

Next, we assessed the subcellular location of PINK1 and hTERT expression. After transfecting HEK293 cells with a plasmid encoding Myc-tagged PINK1 alone or with HA-tagged hTERT, the resulting cell lysates were fractionated into the cytosolic and membrane organelle components. Each sample was then immunoprecipitated with anti-Myc antibody, followed by immunoblotting with anti-HA antiserum. The results revealed that both PINK1 and hTERT were localized in the cytosolic and membrane organelle fractions, but that the binding of PINK1 to hTERT occurred primarily in the membrane organelle fraction (Fig. 1e). Taken together, these data suggest that PINK1 specifically binds to hTERT in mammalian cells, and that the binding primarily occurs within the membrane organelle fraction.

### hTERT suppresses cytosolic PINK1 processing and maintains its location in the mitochondria

**Fig. 2:**
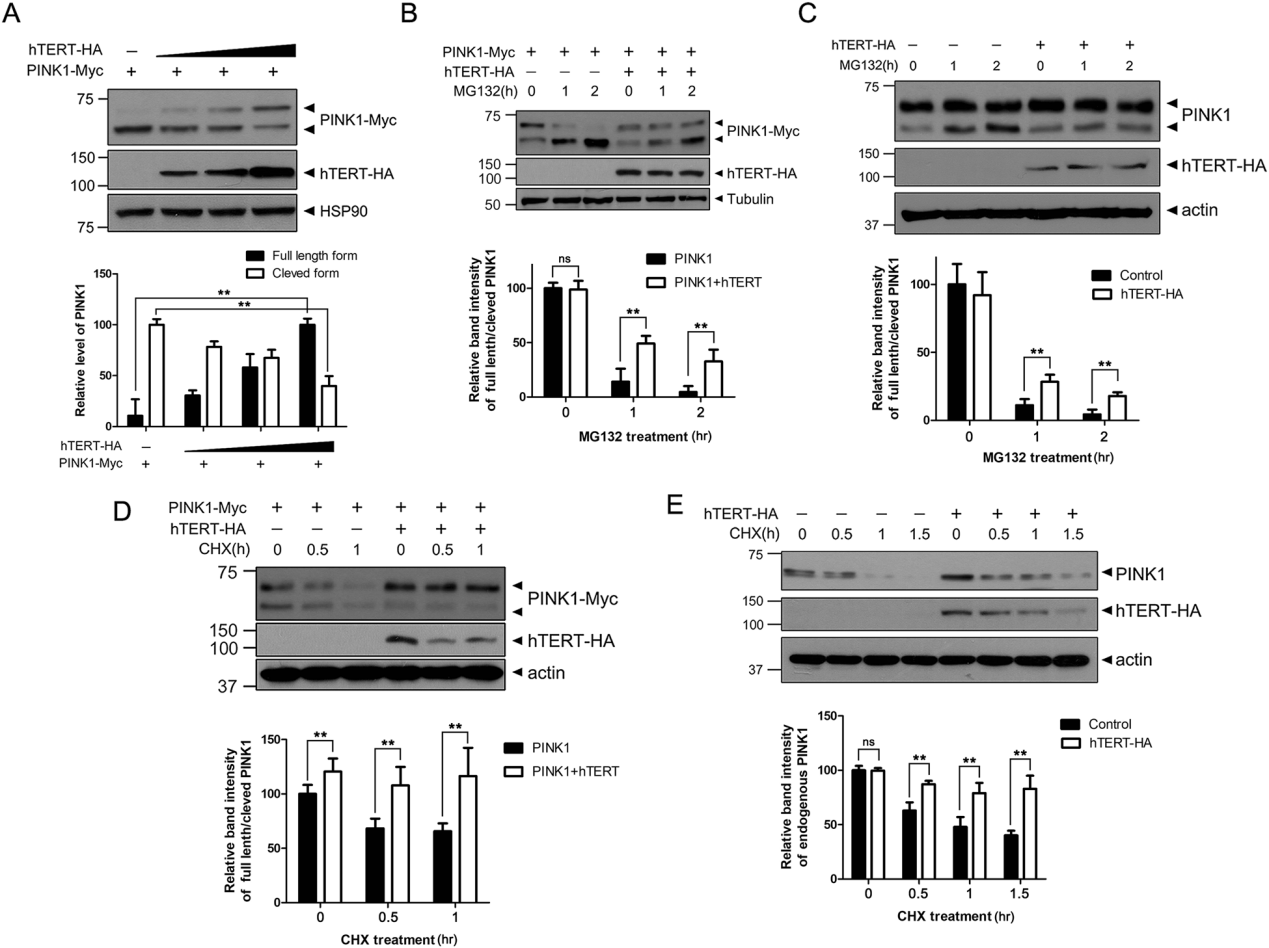
hTERT decreases PINK1 processing. **a** HEK293 cells were transfected for 48 h with a plasmid encoding PINK1-Myc alone or with increasing amounts of plasmid encoding hTERT-HA, and the resulting cell lysates were immunoblotted with the indicated antibodies. **b** HEK293 cells were transfected for 48 h with a plasmid encoding PINK1-Myc alone or with a plasmid encoding hTERT-HA. The cells were treated with 10 μM MG132 for the indicated times and the resulting cell lysates were immunoblotted with the indicated antibodies. **c** HEK293 cells were mock-transfected or transfected with a plasmid encoding hTERT-HA. The cells were treated with 10 μM MG132 for the indicated times and the resulting cell lysates were immunoblotted with the indicated antibodies. **d** HEK293 cells were transfected for 48 h with a plasmid encoding PINK1-Myc alone or with a plasmid encoding hTERT-HA. The cells were treated with 20 μg/ml cycloheximide for the indicated times and the resulting cell lysates were immunoblotted with the indicated antibodies. **e** HEK293 cells were mock-transfected or transfected with a plasmid encoding hTERT-HA. The cells were treated with 20 μg/ml cycloheximide for the indicated times and the resulting cell lysates were immunoblotted with the indicated antibodies. All graph data are presented as the mean ± SEM of three independent experiments (**p ≤ 0.01; **a**–**e**). Statistical analyses were performed using the IBM SPSS Statistics software (version 23.0).

Interestingly, the overexpression of hTERT caused a significant increase in the level of full-length PINK1 in the membrane organelle fraction compared with mock-transfected control cells (Fig. 1e). Because the presence of wild-type hTERT corresponded to a decrease in the levels of the cleaved form of PINK1 in the membrane organelle fraction (Fig. 1e), we hypothesized that hTERT decreases PINK1 processing, resulting in the accumulation of the full-length form of PINK1 at the membrane organelle. To test our theory, we transfected cells with a plasmid encoding PINK1-Myc alone or with increasing amounts of hTERT-HA. Immunoblotting of the cell lysates revealed that the cleavage of exogenous PINK1 is markedly reduced by exogenous hTERT in a dose-dependent manner (Fig. 2a). In addition, the reduction in cleaved PINK1 levels by hTERT was not rescued by treatment with the MG132 proteasome inhibitor (Fig. 2b, c), indicating that hTERT does not affect the degradation of PINK1 via the proteasome machinery.

To determine if hTERT regulates the stability of PINK1, the half-life of PINK1 in the presence or absence of hTERT was compared. Evaluations of the half-life of PINK1 following treatment with cycloheximide revealed that hTERT had no effect on the stability of PINK1, which was in accord with previous results. Regardless of the presence of hTERT, the levels of cleaved PINK1 rapidly decreased (Fig. 2d). However, the concentration of full-length PINK1 was markedly accumulated in the presence of hTERT (Fig. 2d, e). Because full- length PINK1 is not degraded before it is cleaved, these findings suggest that hTERT modulates the cleavage of PINK1 but not its stability.

**Fig. 3:**
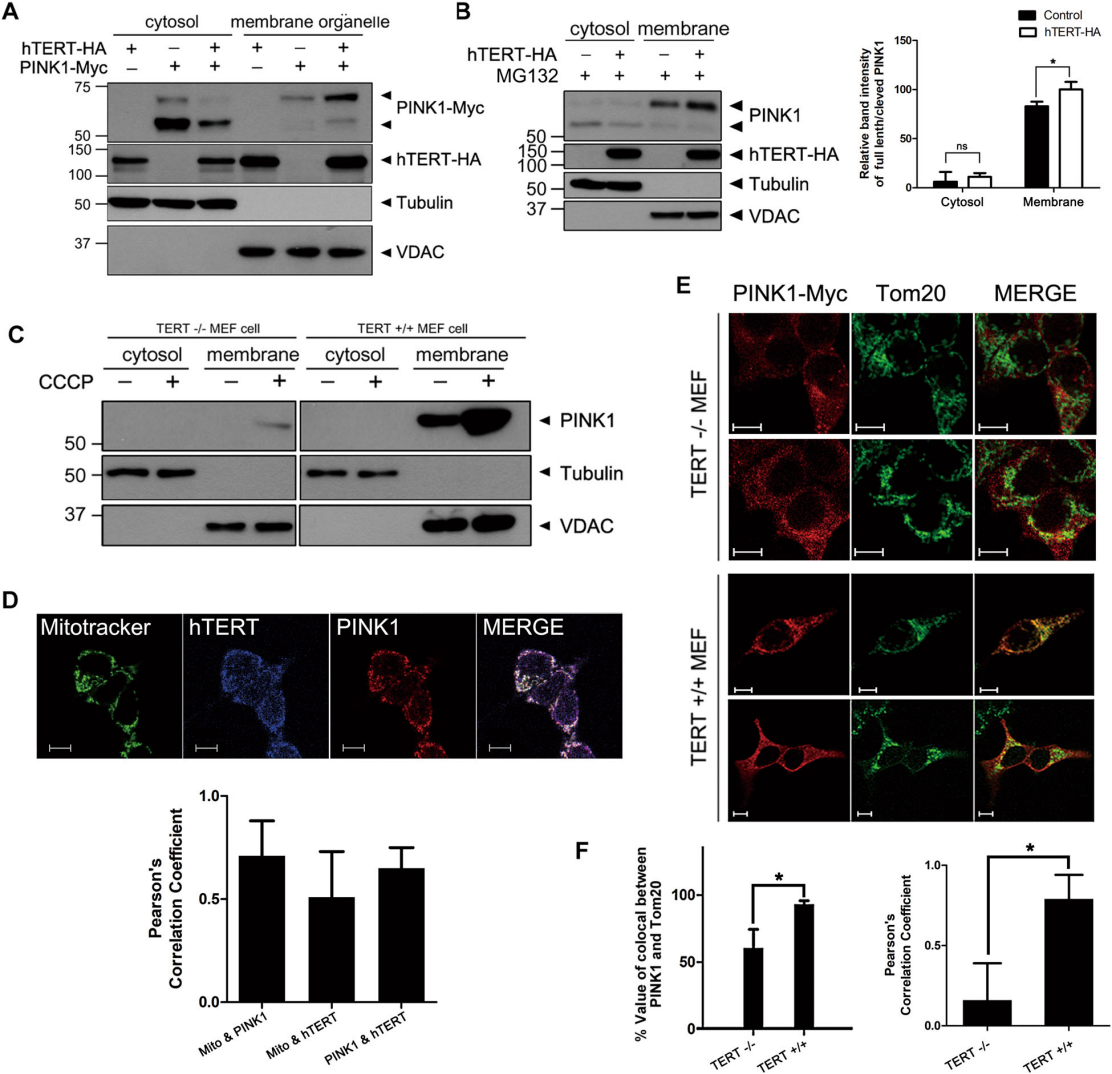
hTERT alters the location of PINK1 to the mitochondria. **a**, **b** HEK293 cells were transfected for 48 h with a plasmid encoding hTERT-HA (**b**) and/or Myc-PINK1 (**a**). The resulting cell lysates were separated into cytosolic and membrane organelle fractions. Tubulin and VDAC served as markers for the cytosolic and the mitochondrial fractions, respectively. Data are presented as the mean ± SEM of three independent experiments (*p ≤ 0.05; **b**). **c** *TERT*+/+and *TERT*−/− MEFs were treated with vehicle or 10 μM CCCP for 4 h. The resulting cell lysates were separated into cytosolic and membrane organelle fractions. **d** Representative confocal images of MitoTracker (green), endogenous PINK1 (red), and hTERT (blue) immunostaining are shown. Scale bar = 10 μm. Pearson′s correlation coefficient of the colocalization between PINK1 and MitoTracker was also analyzed by Image J software. Data are presented as the mean ± SEM of three independent experiments. **e** *TERT*+/+ and *TERT*−/− MEFs expressing Myc-PINK1 were treated with 10 μM CCCP for 4 h before fixation. Representative confocal images of Myc-PINK1 (red) immunostaining. Mitochondria were stained with Tom20 (green). Scale bar = 10 μm. **f** The colocalization of PINK1 and Tom20 was analyzed by calculating Pearson′s correlation coefficient with Image J software. Data are presented as the mean ± SEM of six independent experiments. (***p ≤ 0.001).

Because full-length PINK1 accumulates on the outer membrane of mitochondria and its cleaved form is transported into and located in the cytosol, we investigated if hTERT affects the subcellular localization of PINK1. As shown in Fig. 3a, cells that coexpressed wild-type PINK1 and hTERT exhibited increased accumulation of full-length PINK1 in the membrane organelle fraction compared with cells with PINK1 alone. In addition to ectopic PINK1, the overexpression of hTERT markedly increased the accumulation of the full-length form of endogenous PINK1 in the membrane organelle fraction of MG132 treated cells (Fig. 3b).

CCCP reduces mitochondrial membrane potential, resulting in the loss of mitochondrial membrane integrity and subsequent damage. Consequently, full-length PINK1 was retained in the mitochondria, triggering mitophagy to eliminate the damaged mitochondria. When we compared the levels of full-length PINK1 in *TERT*−/− and control *TERT*+/+ MEFs in the presence or absence of CCCP treatment, two cell types displayed an increase of full-length PINK1, primarily in the membrane fraction. In addition, full-length PINK1 levels were notably enhanced in *TERT*+/+ MEF. Conversely, full-length PINK1 levels in the membrane fraction of *TERT*−/− MEFs were significantly reduced (Fig. 3c). Moreover, immunocytochemical analysis revealed that endogenous PINK1 and hTERT colocalize with the mitochondrial marker MitoTracker with the values of Pearson’s correlation coefficient 0.73 (MitoTracker & PINK1), 0.51 (MitoTracker & hTERT), and 0.63 (PINK1 & hTERT), respectively (Fig. 3d). In addition, the Myc-tagged PINK1 and Tom20 colocalization signal was more pronounced in *TERT*+/+ MEFs with the value of Pearson’s correlation coefficient 0.75, than in *TERT*−/− MEFs having the same value of 0.18 (Fig. 3e, f). These data indicated that hTERT inhibits the cytosolic cleavage of full-length PINK1 and maintains its location within the mitochondria.

Because hTERT levels are known to be remarkably induced by anisomycin^18^, we evaluated the effects of anisomycin treatment on the accumulation of full-length PINK1 in the mitochondria. In accord with prior findings, the results of the immunoblot analysis revealed that the induction of endogenous hTERT in HEK293 cells was increased following treatment with anisomycin (Fig. S1a). Moreover, anisomycin treatment also increased PINK1 levels. Conversely, anisomycin-induced PINK1 levels were markedly reduced when cells were transfected with *hTERT*-siRNA (Fig. S1b), suggesting that hTERT mediates the anisomycin-induced accumulation of PINK1. This theory was further supported by the finding that anisomycin treatment profoundly increased the accumulation of full-length PINK1 levels in *TERT*+/+ MEFs compared with *TERT*−/− MEFs (Fig. S1c, d). As a control, treatment of *TERT*−/− MEFs with anisomycin had no direct effect on the PINK1 level (Fig. S1c). The analysis of cell lysate fractions also revealed that full-length PINK1 accumulation was greater in the membrane organelle following anisomycin treatment and the induction of hTERT (Fig. S1e). Taken together, the results indicate that hTERT decreases PINK1 processing, resulting in the accumulation of its full-length form into the membrane organelle.

### hTERT disrupts the binding of PINK1 to MPPβ, reducing PINK1 processing

**Fig. 4:**
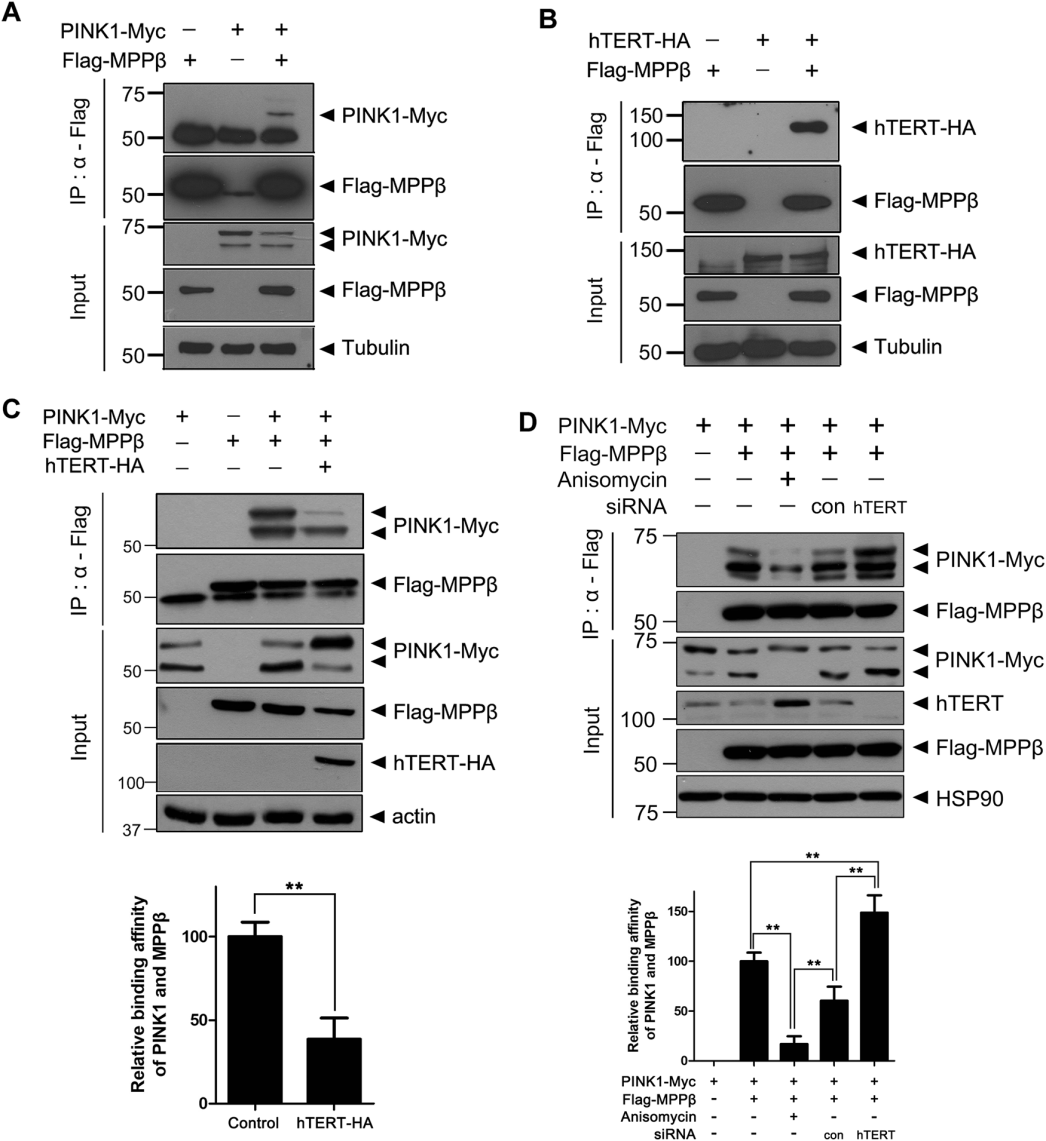
hTERT decreases PINK1-MPPβ binding. **a** HEK293 cells were transfected for 24 h with a plasmid encoding Myc-PINK1 and/or FLAG-MPPβ. The resulting total cell lysates were immunoprecipitated with anti-FLAG antibody and immunoblotted with the indicated antibodies. **b** HEK293 cells were transfected for 48 h with a plasmid encoding hTERT-HA and/or FLAG-MPPβ. The resulting total cell lysates were immunoprecipitated with anti-FLAG antibody and immunoblotted with the indicated antibodies. **c** HEK293 cells were transfected for 48 h with a plasmid encoding PINK1-Myc, FLAG-MPPβ, or hTERT-HA alone or in combination. The resulting cell lysates were immunoprecipitated with anti-FLAG antibody and immunoblotted with the indicated antibodies. **d** HEK293 cells were transfected with plasmid encoding PINK1-Myc, FLAG-MPPβ, or *hTERT*-siRNA alone or in combination. After 48 h, the cells were treated with vehicle or 40 μM anisomycin for 30 h. The resulting cell lysates were immunoprecipitated with anti-FLAG antibody and immunoblotted with the indicated antibodies. The graph data are presented as the mean ± SEM of three independent experiments (**p ≤ 0.01; **c**, **d**).

To determine the mechanism underlying hTERT-mediated inhibition of mitochondrial PINK1 processing, we tested whether hTERT regulates the action of the mitochondrial proteases involved in PINK1 processing. It is known that PINK1 undergoes proteolytic cleavages through the actions of MPP and the rhomboid protease PARL^5^. When we examined whether PINK1 actually binds to MPP as a control, the co-IP analysis revealed that the catalytic subunit of MPP (MPPβ) physically binds to PINK1 (Fig. 4a). The results of the co-IP analysis also demonstrated that ectopically expressed MPPβ binds to hTERT in HEK293 cells (Fig. 4b). In addition, the interaction between PINK1 and MPPβ was notably decreased by hTERT overexpression (Fig. 4c). Furthermore, the binding of PINK1 to MPPβ was markedly reduced in HEK293 cells treated with anisomycin following the induction of hTERT (Fig. 4d). These data suggest that hTERT decreases mitochondrial PINK1 processing by reducing the binding of PINK1 to MPPβ.

### hTERT increases autophagy and mitophagy marker levels

**Fig. 5:**
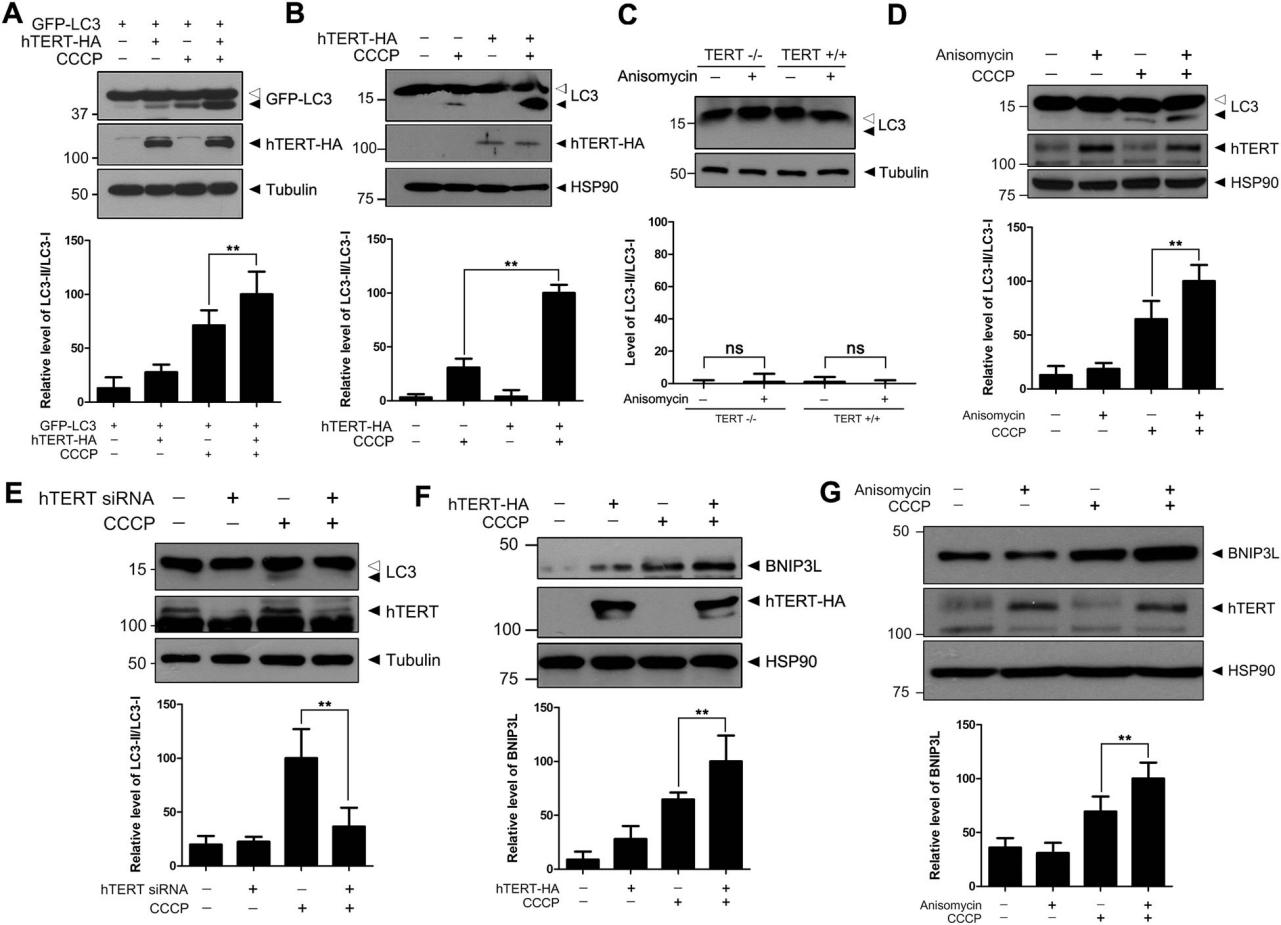
hTERT increases the expression of the autophagy marker LC3 and the mitophagy marker BNIP3L. **a**, **b** HEK293 cells were transfected with a plasmid encoding hTERT-HA (**b**) and GFP-LC3 (**a**). After 48 h, the cells were treated with 10 μM CCCP for 4 h. The resulting cell lysates were immunoblotted with the indicated antibodies. **c** *TERT*+/+and *TERT*−/− MEFs were treated with 40 μM anisomycin for 30 h and the resulting cell lysates were immunoblotted with the indicated antibodies. **d**, **e** HEK293 cells were transfected with a plasmid encoding GFP-LC3. After 24 h, the cells were transfected with *hTERT*-siRNA (**e**) or treated with 40 μM anisomycin (**d**). The resulting cell lysates were immunoblotted with the indicated antibodies. **f**, **g** HEK293 cells were transfected with a plasmid encoding hTERT-HA (**f**) or treated with 40 μM anisomycin (**g**). The resulting cell lysates were immunoblotted with the indicated antibodies. All graph data are presented as the mean ± SEM of three independent experiments (**p ≤ 0.01; **a**–**g**).

As previously noted, the accumulation of full-length PINK1 in mitochondria and its subsequent induction of mitophagy under CCCP-induced mitochondrial damage has been established^20^. Hence, we sought to determine whether the hTERT-mediated increase in full-length PINK1 corresponded to efficient management of mitophagy rates. After cells were transfected with a plasmid encoding wild-type hTERT and/or treated with CCCP, immunoblot analysis of cell lysate revealed that CCCP treatment increased the second form of the autophagy marker LC3 (LC3-II), and its accumulation was even greater with hTERT overexpression (Fig. 5a, b). As a control, CCCP treatment had no effect on the hTERT level (Fig. 5a). Also, hTERT overexpression or anisomycin treatment did not affect endogenous LC3-II accumulation (Fig. 5b, c).

When the cells were treated with either CCCP or anisomycin alone or in combination, the accumulation of full-length PINK1 was similar (Fig. 5d) to the increase apparent in Fig. 5a, b. The observed accumulation of LC3-II was synergistic following combined CCCP and anisomycin treatment (Fig. 5d). Conversely, LC3-II levels were markedly inhibited by the siRNA-mediated knockdown of endogenous hTERT (Fig. 5e). These data suggest that the anisomycin-induced accumulation of LC3-II occurs through hTERT induction, which was consistent with our previous results (Fig. S1a, b; Fig. 5d). The BNIP3L protein was another commonly used marker of mitophagy ^21^. Similar to the effect on LC3-II, the results of immunoblot assays of cell lysates treated with CCCP or transfected with hTERT showed that the addition of hTERT or CCCP increased endogenous BNIP3L levels in an additive way. (Fig. 5f). Treatment of anisomycin alone or in combination with CCCP also had a similar effect on BNIP3L levels (Fig. 5g).

Next, we investigated whether PINK1 or increased PINK1 levels were necessary for the observed increase in mitophagy markers in response to hTERT. The amount of intracellular LC3-II was increased after CCCP treatment in both *PINK1*+/+ and *PINK1*−/− MEFs (Fig. S2a); although, the observed increase in LC3-II were higher in *PINK1*+/+ MEFs compared with *PINK1*−/− MEF (Fig. S2a). Additionally, although hTERT plus CCCP triggered increases in LC3-II in *PINK1*+/+ MEFs (Fig. S2c), no increase was observed in *PINK1*−/− MEFs (Fig. S2b). Interestingly, transfecting *PINK1*+/+ MEFs with hTERT did not yield considerable increase in LC3-II levels in the absence of CCCP treatment. These results suggest that the hTERT-mediated accumulation of PINK1 is not absolutely required for the observed CCCP-induced increase in the mitophagy marker, but it stimulates the induction of LC3-II following CCCP treatment. Collectively, these data suggest that hTERT increases autophagy and mitophagy marker levels by mediating the accumulation of PINK1.

### hTERT increases the interaction of PINK1 with Tom20 and parkin after CCCP treatment

**Fig. 6:**
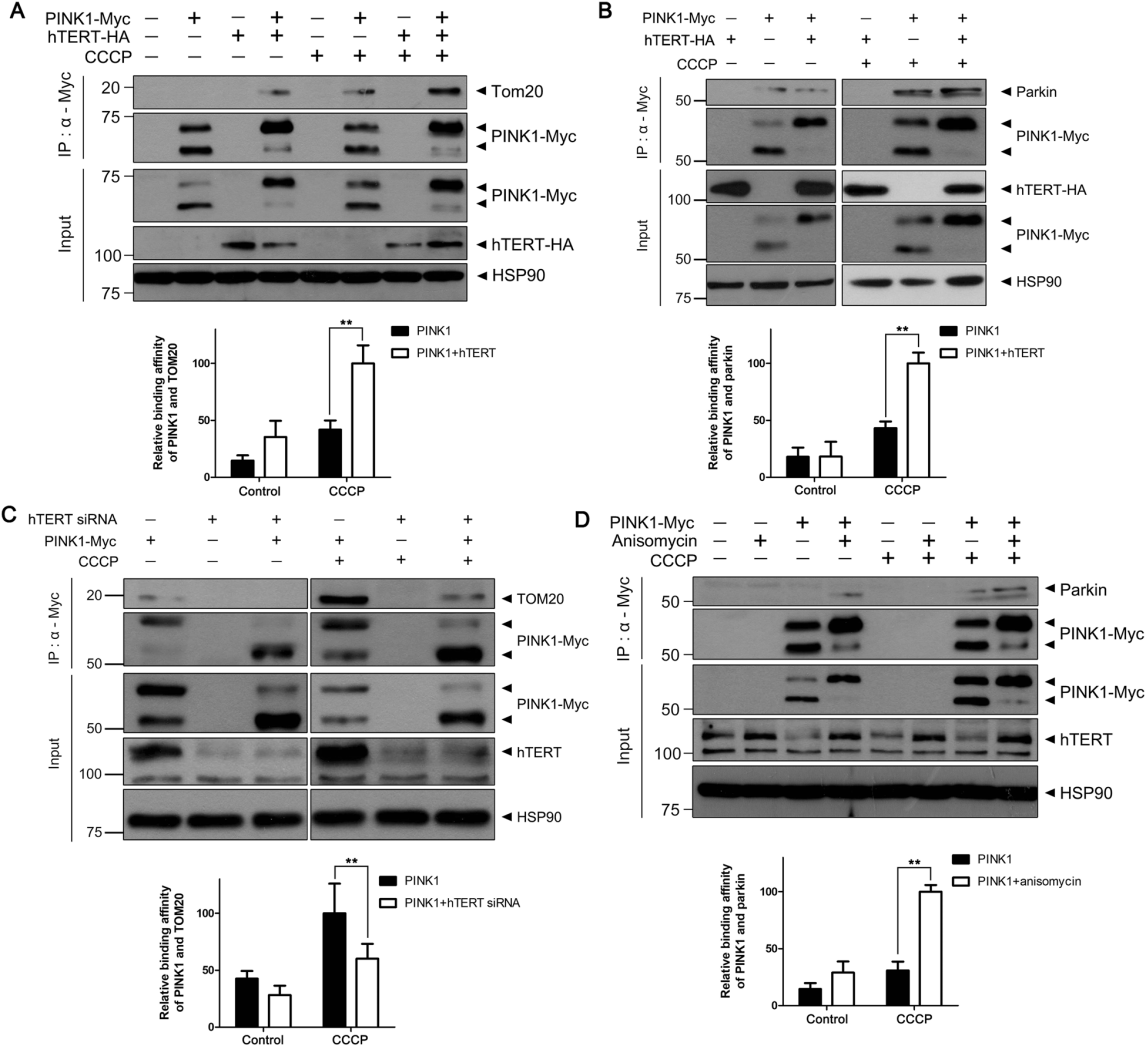
hTERT increases the interaction of PINK1 with Tom20 and parkin after treatment with CCCP. **a**–**c** HEK293 cells were transfected with a plasmid encoding Myc-PINK1 and hTERT-HA (**a**, **b**) or a plasmid encoding Myc-PINK1 and *hTERT*-siRNA (**c**). After 48 h, the cells were treated for 4 h with 10 μM CCCP. The resulting cell lysates were immunoblotted with the indicated antibodies. **d** HEK293 cells were mock-transfected or transfected with Myc-PINK1. After 24 h, the cells were treated for 48 h with 40 μM anisomycin followed by 10 μM CCCP for 4 h. The resulting cell lysates were immunoblotted with the indicated antibodies. All graph data are presented as the mean ± SEM of three independent experiments (**p ≤ 0.01; **a**–**d**).

When mitophagy is induced by mitochondrial damage (i.e., triggered by CCCP treatment), PINK1 levels are increased and PINK1 subsequently binds to Tom20 and recruits and increases the interaction with parkin^4^. To determine whether hTERT affected the binding of PINK1 to these proteins, we performed co-IP analyses. Immunoblotting assays revealed that the overexpression of hTERT triggers the increased binding of PINK1 to Tom20 (Fig. 6a) and parkin (Fig. 6b) after CCCP treatment. In addition, the increased binding of PINK1 to Tom20 after CCCP treatment was markedly inhibited by siRNA-mediated knockdown of endogenous hTERT (Fig. 6c). While anisomycin treatment caused increase in hTERT, it did not result in the binding of PINK1 to parkin. However, the combined treatment of CCCP and anisomycin markedly increased the binding of PINK1 to parkin compared with cells treated with CCCP alone (Fig. 6d). Taken together, these data suggest that hTERT increases the binding of PINK1 to Tom20 and parkin following treatment with CCCP.

### hTERT induces mitophagy and recovers mitochondrial functions

**Fig. 7:**
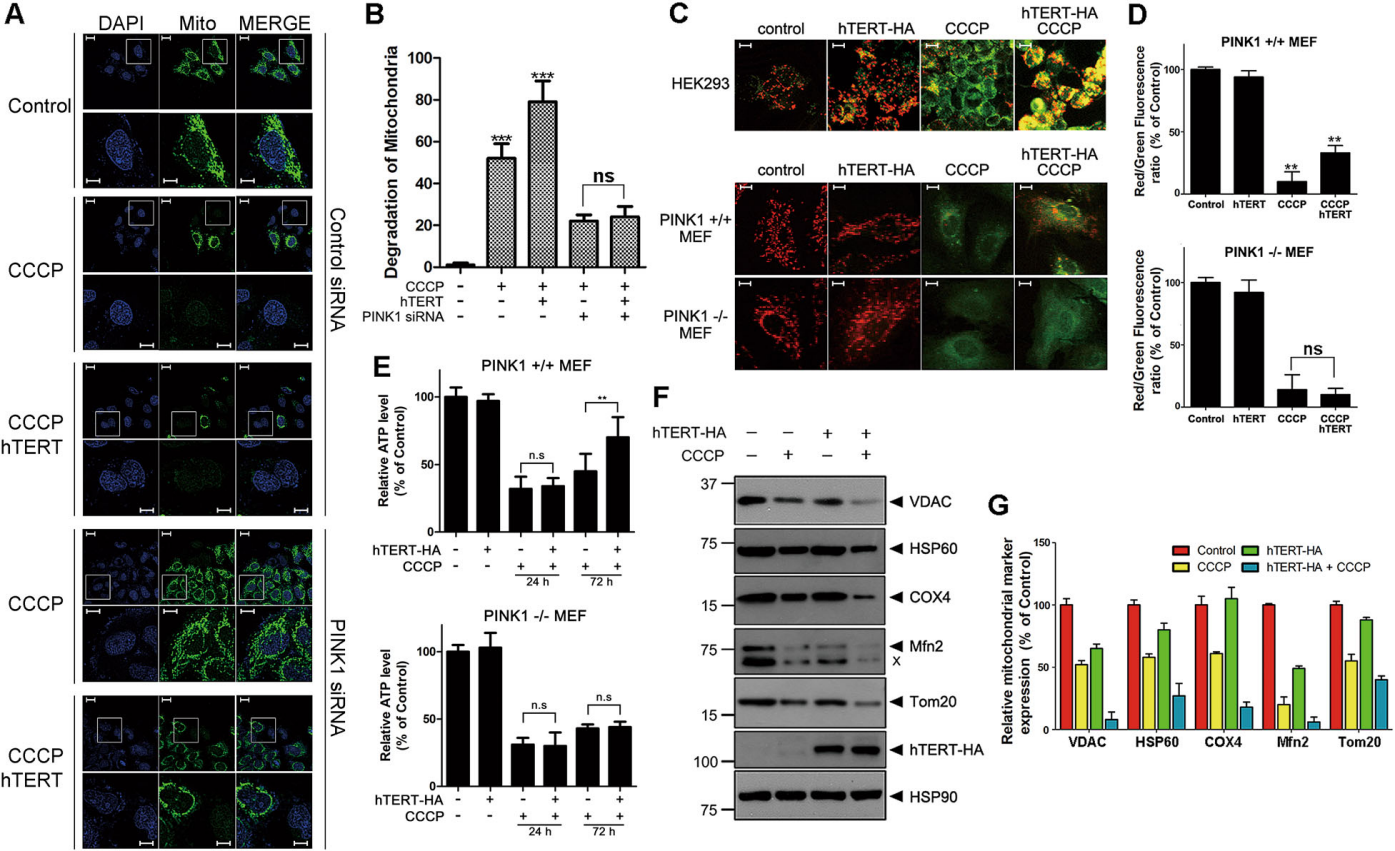
hTERT promotes CCCP-induced mitophagy. **a** Where indicated, HeLa cells overexpressing wild-type parkin were transfected with wild-type hTERT, scrambled siRNA (control siRNA), or/and *PINK1*-siRNA for 24 h and treated with either DMSO (control) or 20 μM CCCP for an additional 24 h. Mitochondrial degradation was assessed in each cell type using the MitoTracker mitochondria-selective probe (green) and DAPI (blue). Second row shows enlarged views of the white boxed area of the first row. Scale bar = 10 μm for the upper panel; 5 μm for the bottom enlarged image. **b** Mitochondrial loss in each cell type was quantified. Data are presented as the mean ± SEM of three independent experiments (***p ≤ 0.001), and ~100 cells were analyzed per experiment. **c** Where specified, HEK293 cells, *PINK1*+/+MEF, or *PINK1*−/− MEFs were mock-transfected or transfected for 24 h with hTERT-HA and treated with DMSO or 20 μM CCCP for 4 h. Mitochondrial membrane potential (MMP) of each sample was then detected by JC-1 staining. Scale bars = 20 μm for the upper panel from the top; 5 μm for the middle and bottom image. **d** The red/green fluorescence ratio in the *PINK1*+/+and *PINK1*−/− MEFs in **c** was quantified. Error bars represent standard deviations of three independent experiments, and ~100 cells were analyzed per experiment. **e** Where specified, *PINK1*+/+ and *PINK1*−/− MEFs were mock-transfected or transfected with hTERT-HA for 24 h, followed by treatment with DMSO or 20 μM CCCP for the indicated times. The ATP level of each sample was then measured using an ATP determination kit. **f** Neuroblastoma SH-SY5Y cells were either mock-transfected or transfected with hTERT-HA for 24 h, and treated with 10 μM CCCP for 4 h. The resulting cell lysates were immunoblotted with the indicated antibodies. **g** Relative band intensities of each mitochondrial marker protein were measured using the MultiGauge V. 3.1 program. Error bars represent standard deviations of three independent experiments.

Next, we examined if hTERT actually induces mitophagy and whether the removal of damaged mitochondria restored mitochondrial function. To explore this, mitophagy rates in control and hTERT-overexpressing cells were compared. After CCCP treatment, HeLa cells overexpressing parkin exhibited considerable loss of mitochondria via mitophagy. In addition, hTERT overexpression caused greater mitophagy rates, but this phenotype was not observed in *PINK1*-knockdown cells (Fig. 7a, b). When we measured change in the mitochondrial membrane potential using the mitochondrial specific JC-1 dye, the overexpression of hTERT alone did not significantly perturb the mitochondrial membrane potential (Fig. 7c, d). However, CCCP treatment as a control caused a considerable loss in membrane potential, as expected. Further, hTERT overexpression rescued the CCCP-induced loss of mitochondrial membrane potential in HEK293 cells (Fig. 7c). Moreover, the effect of hTERT was seen in *PINK1*+/+ MEFs, but not in *PINK1*−/− MEFs (Fig. 7c, d). We also measured intracellular ATP levels to serve as another index of mitochondrial function in the presence or absence of hTERT. As expected, intracellular ATP levels were unaffected by hTERT alone, but CCCP treatment greatly reduced ATP levels (Fig. 7e). Moreover, the CCCP-induced reduction in intracellular ATP levels was rescued by hTERT at 72 h after CCCP treatment, while it was initially unaffected within 24 h (Fig. 7e). Since the removal of damaged mitochondria is integrated to mitochondrial biogenesis, it can be expected that the increase of mitophagy by hTERT would promote the mitochondrial biogenesis, enhancing ATP production after 72 h of treatment with CCCP. However, the effect of hTERT on intracellular ATP production was not observed in *PINK1*−/− MEFs (Fig. 7e). Finally, we examined changes in the levels of typical mitochondrial proteins in the presence or absence of hTERT. Immunoblot analyses of cell lysates with anti-VDAC antiserum revealed that CCCP treatment reduced VDAC levels following CCCP treatment, primarily due to increased mitophagy (Fig. 7f, g). While hTERT alone do not significantly affect VDAC levels, transfection of hTERT plus CCCP treatment caused a decrease in mitochondrial protein levels that was much greater than changes induced by CCCP treatment alone (Fig. 7f, g). The same pattern of change was also observed in other mitochondrial proteins (i.e., Hsp60, Cox4, Mfn2, and Tom20) (Fig. 7f, g). Taken together, these data suggest that hTERT acts as a novel controller of PINK1 processing, leading to an increase in mitophagy in response to CCCP-induced mitochondrial damage.

## Discussion

Mitochondrial autophagy, or mitophagy, regulates mitochondrial numbers and protects cells from the deleterious effects of mitotoxicity, serving as the major pathway for maintaining mitochondrial network homeostasis. A mitochondrial targeting signal results in the primary localization of PINK1 in the mitochondria, where it affects mitochondrial homeostasis and dynamics, including mitophagy, bioenergetics, fission, and fusion^3^. PINK1 is also detected in the cytosol, however, where it protects cells against various cytotoxic agents and regulates dendritic morphogenesis in neurons^22,23^. Under steady-state conditions, PINK1 is constitutively imported into the mitochondria, where it is cleaved and degraded by the ubiquitin-proteasome system (UPS). The proteolysis of PINK1 is mediated by the outer and inner mitochondrial membrane-associated MPP and the PARL protease. Based on the findings that PINK1 is involved in a variety of physiological processes, its proteolysis should be tightly regulated by its binding proteins or modulators. As well, its dysfunction contributes to the progression of a diverse number of diseases, including PD. Following the loss of mitochondrial membrane potential, which can be artificially induced by mitochondrial uncouplers (e.g. CCCP), PINK1 is stabilized and activated on the outer mitochondrial membrane, leading to the recruitment of cytosolic parkin to damaged mitochondria, where it is activated.

Regarding the link between hTERT and the mitochondria, several reports indicated the mitochondrial location and the regulatory effect of hTERT. For example, under mild stress and ROS production, nuclear hTERT is targeted to the mitochondria by an N-terminal leader sequence^14,17^. Within the mitochondria hTERT reduces intracellular ROS production, improves mitochondrial respiratory activity and function, and inhibits ROS-induced cell death^13,14^. In addition, hTERT inhibits cytosolic acidification, the mitochondrial translocation of Bax, the decrease in mitochondrial transmembrane potential, the release of cytochrome C to the cytosol, and cell death^14^. Mitochondrial TERT also exerts a novel protective function by binding to mitochondrial DNA, protecting it against oxidative stress-induced damage^24^. Furthermore, inhibiting hTERT expression induces apoptosis through a mitochondrial-dependent pathway^25^. Similarly, several lines of evidence indicate that TERT suppresses the mitochondrial death pathway^26,27^. For example, TERT protects against Bax-activating staurosporine in both 293T cells and MEFs^28^. Furthermore, the death of neonatal motor neurons after axotomy is mediated by Bax and the subsequent mitochondrial apoptotic cascades^29,30^. In addition to the prior reports, the present data provides additional evidence that hTERT could regulate mitochondrial function and its quality control processes by regulating PINK1.

As previously described, proper mitochondrial function and energy metabolism are critical to very complicated neuronal functions, and mitochondrial dysfunction largely contributes to neuronal cell death. Because hTERT also affects mitochondrial function in direct and indirect ways^14^ and associated with neuronal death, it is possible that hTERT also affects the occurrence and progression of PD. In accord with this theory, the present study provided evidence of a close link between hTERT and PD pathogenesis via the positive modulation of PD-associated PINK1 processing and subsequent mitophagy.

MPP and PARL, the most well-known proteases involved in PINK1 processing^5^, bind directly to PINK1, triggering the cleavage of PINK1 after it enters the mitochondria via the TOM and TIM complexes. It is also known that mutations in these proteins prevent the proper processing of mitochondrial proteins affecting the onset of mitochondrial diseases^31^. However, the factors that regulate the binding of PINK1 to these proteins remain unclear. To this end, the current study demonstrated that hTERT binds directly to the catalytic β subunit of MPP, inhibiting its proteolytic processing of PINK1 within the mitochondrial outer membrane and resulting in the accumulation of full-length PINK1. In turn, increased levels of full-length PINK1 in the mitochondria play an important role in the PINK1/parkin-dependent pathway. PINK1 recruits parkin from the cytoplasm to the mitochondria and the E3 ligase parkin then ubiquitinates target protein, subsequently recruiting autophagy adapters and LC3-II to the mitochondria^32^. The current findings demonstrated that hTERT markedly increases the formation of LC3-II, which is mediated by the regulation of PINK1.

Based on the previous findings that (1) oxidative stress is the most important factor that controls the localization of PINK1 to mitochondria^33^, (2) PINK1 regulates mitochondrial quality control, including mitophagy and the fission/fusion process^34^, and (3) hTERT alleviates ROS-induced damage to mitochondria^17^, we hypothesized that PINK1 and hTERT played protective roles in mitochondrial function and could be biochemically or/and functionally linked. Our hypothesis was verified by the finding that hTERT positively regulates mitophagy by inhibiting cytosolic PINK1 processing. These data are further supported by other reports that suggest important roles of hTERT in protecting neuronal cells from cell death both in vitro and in vivo^35,36^. For example, TERT overexpression protected pheochromocytoma cells from apoptosis induced by trophic factor withdrawal and amyloid peptides^37,38^. Conversely, TERT suppression increased the sensitivity of primary neuronal cells to excitotoxins as well as amyloid peptides^37,38^. The transgenic overexpression of hTERT reportedly confers resistance to ischemic brain injury and NMDA receptor-mediated neurotoxicity in the mouse brain^39^. In addition, hTERT-deficient neurons isolated from the embryos are more sensitive to NMDA than those from wild-type ones^28^. TERT-deficient mice also exhibited significantly altered anxiety-like behaviors and abnormal olfaction following measurements of the hippocampus and the olfactory bulb functions, respectively^40^.

Because PINK1 functions in a pro-survival pathway, its activity is presumably tightly regulated upstream. However, only a few studies have focused on the mechanism underlying the modulation of steady-state PINK1 levels and the identification of the factor(s) involved. For example, it is known that the UPS regulates PINK1 stability. Specifically, the TRAF6-mediated Lys63-linked ubiquitination of PINK1 is required for PINK1 stabilization on damaged mitochondria^41^. Moreover, the cleaved form of PINK1 is a target of the N-end rule pathway, a component of the UPS that mediates its rapid turnover^42^. Recently, we demonstrated that CHIP acts as a novel E3 ligase of PINK1, promoting its ubiquitination and subsequent proteasome degradation, and ultimately resulting in cytotoxicity by reducing the cytoprotective effect of PINK1^43^. In addition to UPS, BAG2, BAG5, and Hsp90, a component of the Hsp90/Cdc37 molecular chaperone complex, reportedly regulate PINK1 stability via protein–protein interactions, allowing for the proper mitochondrial translocation of PINK1 and execution of the mitochondrial quality control pathway^16,44,45^. In particular, BAG5 protects cells against 1-methyl-4-phenylpyridinium- and rotenone-induced oxidative damage and mitochondrial dysfunction by up-regulating PINK1^45^. PINK1 also forms a complex with SARM1 and TRAF6, which is important for importing PINK1 to the outer membrane and the stabilization of PINK1 on depolarized mitochondria^41^. Considering the relationship between hTERT and heat shock proteins, the chaperone complex including Hsp70, Hsp90, and p23, is also critical to the correct assembly of telomerase and its proper function^15,46,47^.

In conclusion, the present study proposes a novel pathway for the processing and localization of PINK1. Further, our findings demonstrate that the hTERT-mediated accumulation of PINK1 on mitochondria could increase mitophagy and that altering the activity or regulation of PINK1 might cause or be associated with the progression of PD.

## Supplementary information

Supplementary Figure Legends

Supplementary Figure S1

Supplementary Figure S2
