# Supplementary material for: Human telomerase reverse transcriptase positively regulates mitophagy by inhibiting the processing and cytoplasmic release of mitochondrial PINK1
Source: Cell Death Dis. 2020 Jun 8;11(6):425. doi: 10.1038/s41419-020-2641-7 (PMC7280311; doi:10.1038/s41419-020-2641-7)
Supplement: Supplementary file 1 — Supplementary Figure Legends [file 41419_2020_2641_MOESM1_ESM.docx]

**Supplementary Figure Legends**

**Supplementary Figure S1. Anisomycin-induced hTERT accumulation decreases PINK1 processing and alters its localization to the membrane organelle fraction.** **a** HEK293 cells were treated with 40 μM anisomycin for the indicated times and the resulting cell lysates were immunoblotted with the indicated antibodies. **b** HEK293 cells were mock-transfected or transfected with *hTERT*-siRNA. After 48 h, cells were treated with 40 μM anisomycin for 30 h and the resulting cell lysates were immunoblotted with the indicated antibodies. **c, d** *TERT*+/+ and *TERT*-/- MEFs were treated with 40 μM anisomycin for 30 h and the resulting cell lysates were immunoblotted with the indicated antibodies. **e** As indicated, HEK293 cells were consecutively treated with 40 μM anisomycin for 30 h, and with 10 μM MG132 for 4 h. The resulting cell lysates were separated into cytosolic and membrane organelle fractions, and the samples were immunoblotted with the indicated antibodies. Tubulin and VDAC served as markers for the cytosolic and the mitochondrial fractions, respectively. The graph data are presented as the mean ± SEM of three independent experiments (***p* ≤ 0.01; **c-e**).

**Supplementary Figure S2. PINK1 is required for hTERT-mediated increase in the second form of autophagy marker LC3.** **a** *PINK1*+/+ and *PINK1*-/- MEFs were treated with 10 μM CCCP for 4 h. The resulting cell lysates were immunoblotted with the indicated antibodies. **b, c** *PINK1*+/+ (**c**) and *PINK1*-/- (**b**) MEFs were transfected with plasmids encoding GFP-LC3 or hTERT-HA alone or in combination for 48 h, and treated 10 μM CCCP for 4 h. The resulting cell lysates were immunoblotted with the indicated antibodies. All graph data are presented as the mean ± SEM of three independent experiments (***p* ≤ 0.01; **a-c**).
