# Supplementary figures and images for: Human telomerase reverse transcriptase positively regulates mitophagy by inhibiting the processing and cytoplasmic release of mitochondrial PINK1
Source: Cell Death Dis. 2020 Jun 8;11(6):425. doi: 10.1038/s41419-020-2641-7 (PMC7280311; doi:10.1038/s41419-020-2641-7)

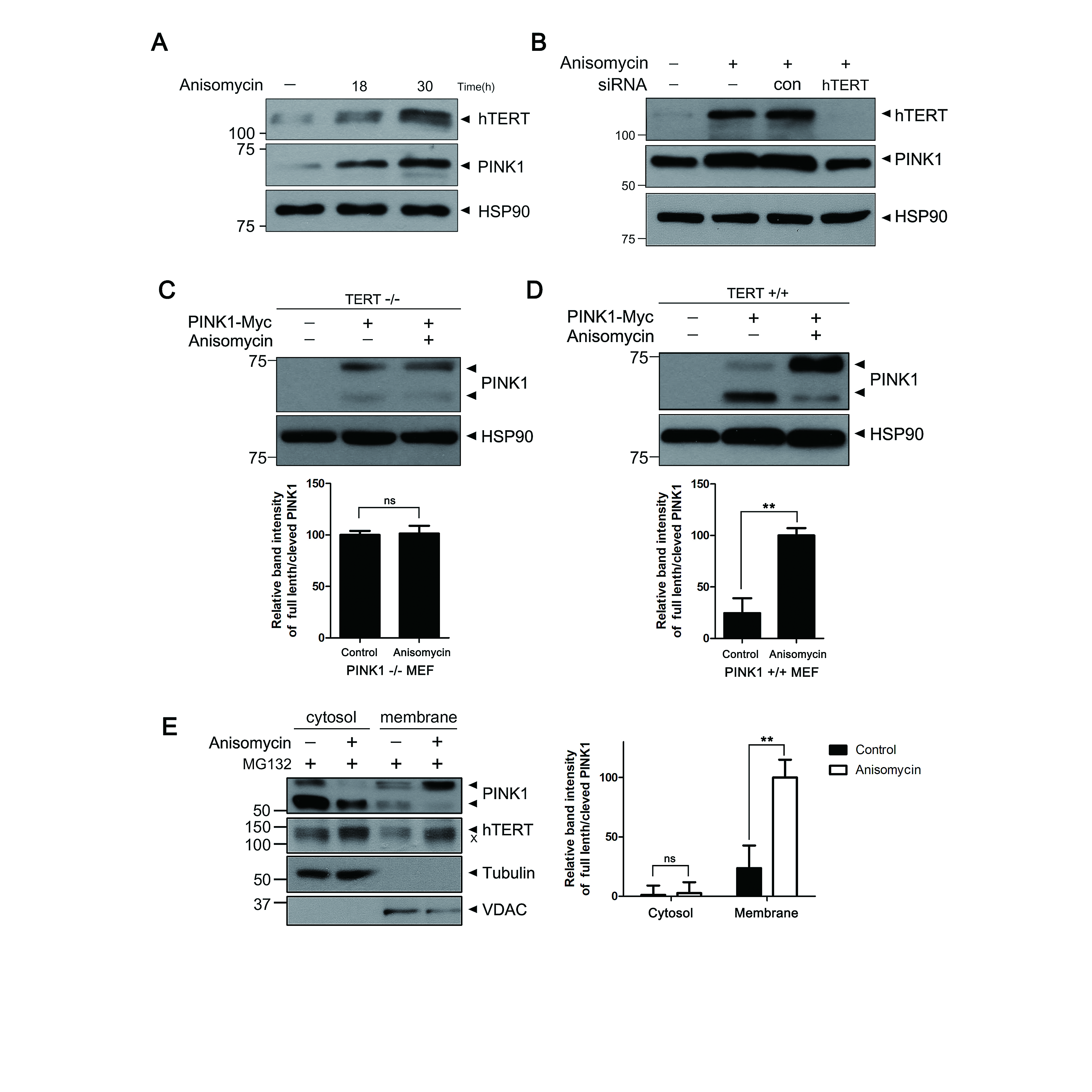

Supplement: Supplementary file 2 — Supplementary Figure S1 [file 41419_2020_2641_MOESM2_ESM.tif]

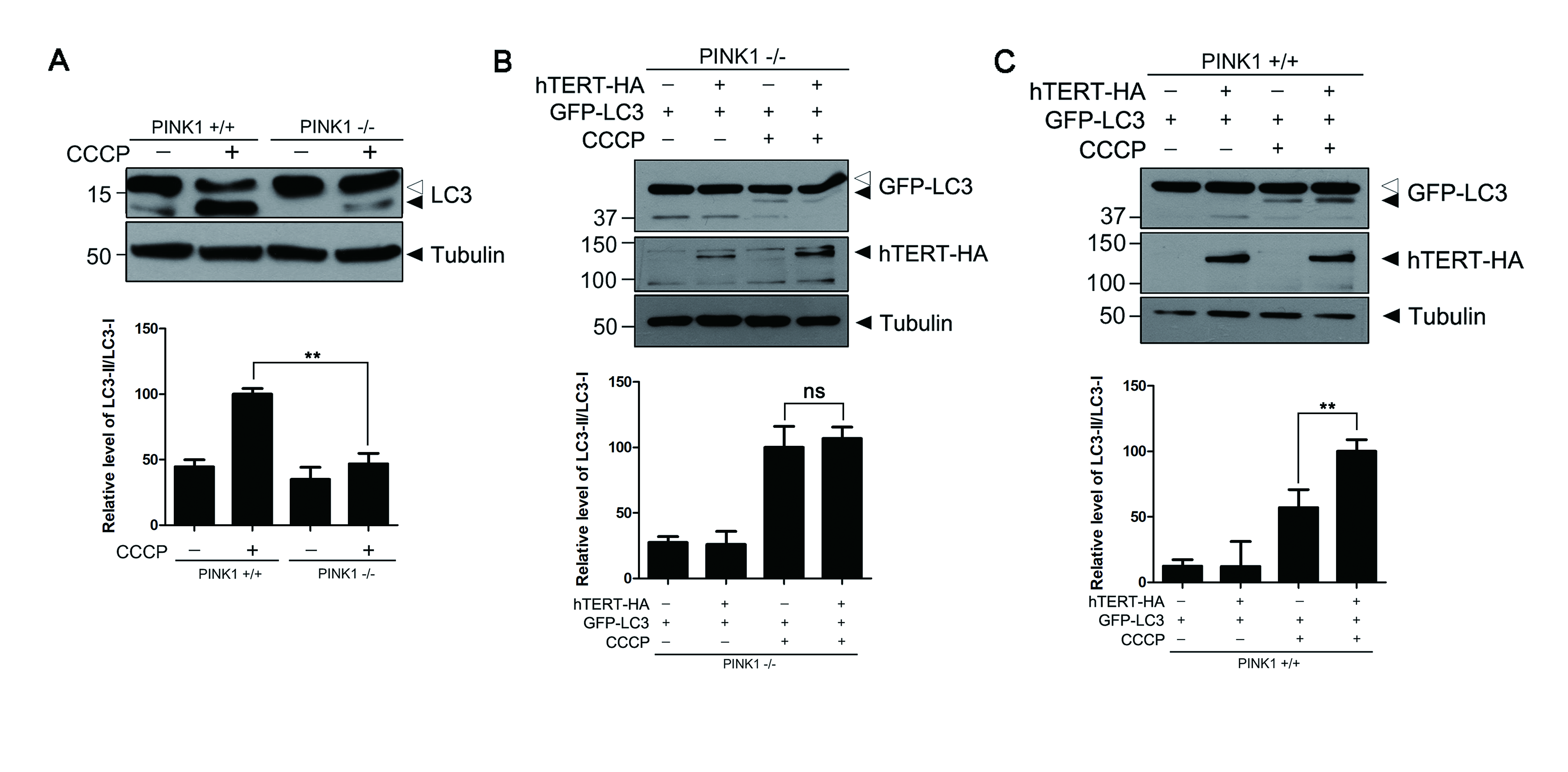

Supplement: Supplementary file 3 — Supplementary Figure S2 [file 41419_2020_2641_MOESM3_ESM.tif]
